# Ecological distribution and genetic diversity of Azolla in Uganda

**DOI:** 10.1186/s12870-023-04146-6

**Published:** 2023-03-08

**Authors:** Nabyonga Lydia, Twaha A. Basamba, Clement Nyakoojo, Abubakar Sadik Mustafa, Ntambi Saidi, Gerald M. Mutumba, Jamilu E. Ssenku

**Affiliations:** 1 Department of Plant Sciences, Microbiology and Biotechnology, College of Natural Sciences, Makerere University, P. O BOX 7062, Kampala, Uganda; 2 Department of Agricultural Production, College of Agriculture and Environmental Studies, Makerere University, P. O BOX 7062, Kampala, Uganda

**Keywords:** Agro-ecological zones, Azolla species, Genetic diversity, Ecological distribution, Wetlands

## Abstract

**Background:**

*Azolla* is an important aquatic fern whose agronomic potential has not been fully exploited in Uganda. This study aimed at determining the genetic variation in the *Azolla* species existing in Uganda and the factors influencing their distribution in the different agro-ecological zones of Uganda. Molecular characterization was preferred in this study because of its efficiency in detecting variations among closely related species.

**Results:**

Four species of *Azolla* were identified in Uganda with 100, 93.36*,* 99.22 and 99.39% sequence identities to the reference database sequences of; *Azolla mexicana*, *Azolla microphylla*, *Azolla filiculoides* and *Azolla cristata,* respectively. These different species were distributed in four out of the ten agro-ecological zones of Uganda which are situated in close vicinity to large water masses. The principal component analysis (PCA) results revealed that maximum rainfall and altitude significantly accounted for the variations in the distribution of *Azolla* with factor loadings of 0.921 and 0.922, respectively.

**Conclusion:**

Massive destruction coupled with prolonged disturbance of *Azolla*’s habitat negatively affected its growth, survival and distribution in the country. Therefore, there is a need to develop standard methods that can preserve the various species of *Azolla*, so as to salvage them for future use, research and reference.

## Background

*Azolla* is a free-floating aquatic fern that is globally distributed. It preferably grows in environments with very slow or non-moving waters, such as moist soils, marshes, swamps, ponds, riverbanks and lakeshores [22]. *Azolla* thrives in a symbiotic association with a nitrogen-fixing cyanobacterium, *Anabaena azollae* [25]. The cyanobacteria provide nitrogen for themselves and their host. In turn, *Azolla* provides a protective environment with already fixed carbon, enabling the cyanobacteria to fix atmospheric nitrogen. This symbiotic characteristic nature of *Azolla* has accorded it tremendous agronomic importance as a bio-fertilizer for crops, especially in soils where nitrogen is limiting. Besides, *Azolla* has a huge potential for use as a livestock feed [4], biofuel [24], the feedstock of bio refineries [5], microbial culture medium [10], and wastewater treatment [3]. Despite its numerous values and thus its dubbing as a “green gold”, by many researchers, its exploitation in Uganda has been curtailed by the lack of knowledge on its identity, ecological distribution and genetic diversity.

According to the classification by the Pteridophyte Phylogeny Group (PPG I), *Azolla* belongs to the family Salviniaceae [21] which comprises seven species. *Azolla* was further divided into two sub-sections (sub-genera), namely Euazolla and Rhizosperma. Euazolla consists of five species including; *Azolla microphylla* Kaulf., *Azolla caroliniana* Willd., *Azolla mexicana* Schlecht.& Cham.ex K.Presl, *Azolla rubra* R.Br., and *Azolla filiculoides* Lam. Sub-section Rhizosperma consists of *Azolla pinnata* R.Br. and *Azolla nilotica* Decne. ex Mett [11, 13, 18–20].

In Uganda, by 1992, only *Azolla pinnata* and *Azolla nilotica* had been identified on Lake Victoria [30]*.* According to [29], *Azolla. pinnata* and *Azolla nilotica* were morphologically identified in Uganda in the regions of Kigezi, West Nile, Lake Kyoga, Lake George, Masaka, Kamuli, Jinja-Tororo highway, Namanve swamp, Kasenyi fishing village (part of Lake Victoria) and Busia. Despite the extensive studies carried out on *Azolla* globally, it is still difficult to identify based on morphological, physiological, and reproductive characteristics [10].

*Azolla* exhibits some phenotypic variations in terms of color and size among other characteristics. However, these variations may not necessarily correspond to variations in their genotypes but rather environmental influences. For instance, high temperatures can elicit stress which usually manifests in terms of color changes from green to reddish brown, following the production of anthocyanins and a reduction in chlorophyll [9, 34]. *Azolla* grows under; temperatures between 18 and 25 °C, pH between 4.5 – 7.5 and humidity of 70-75% and a shady environment with low light intensity [23].

In Uganda, historical records about the distribution and genetic diversity of *Azolla* have been rendered less reliable. This is mainly attributed to the wide spread anthropogenic destruction of its habitats coupled with the less efficient methods of identification that were used in the past. We thus aimed at validating the current ecological and genetic diversity data about *Azolla* in Uganda, so as to pave a way for extensive studies that will contribute to unlocking of its multiple potential uses in the country.

## Results

**Fig. 1:**
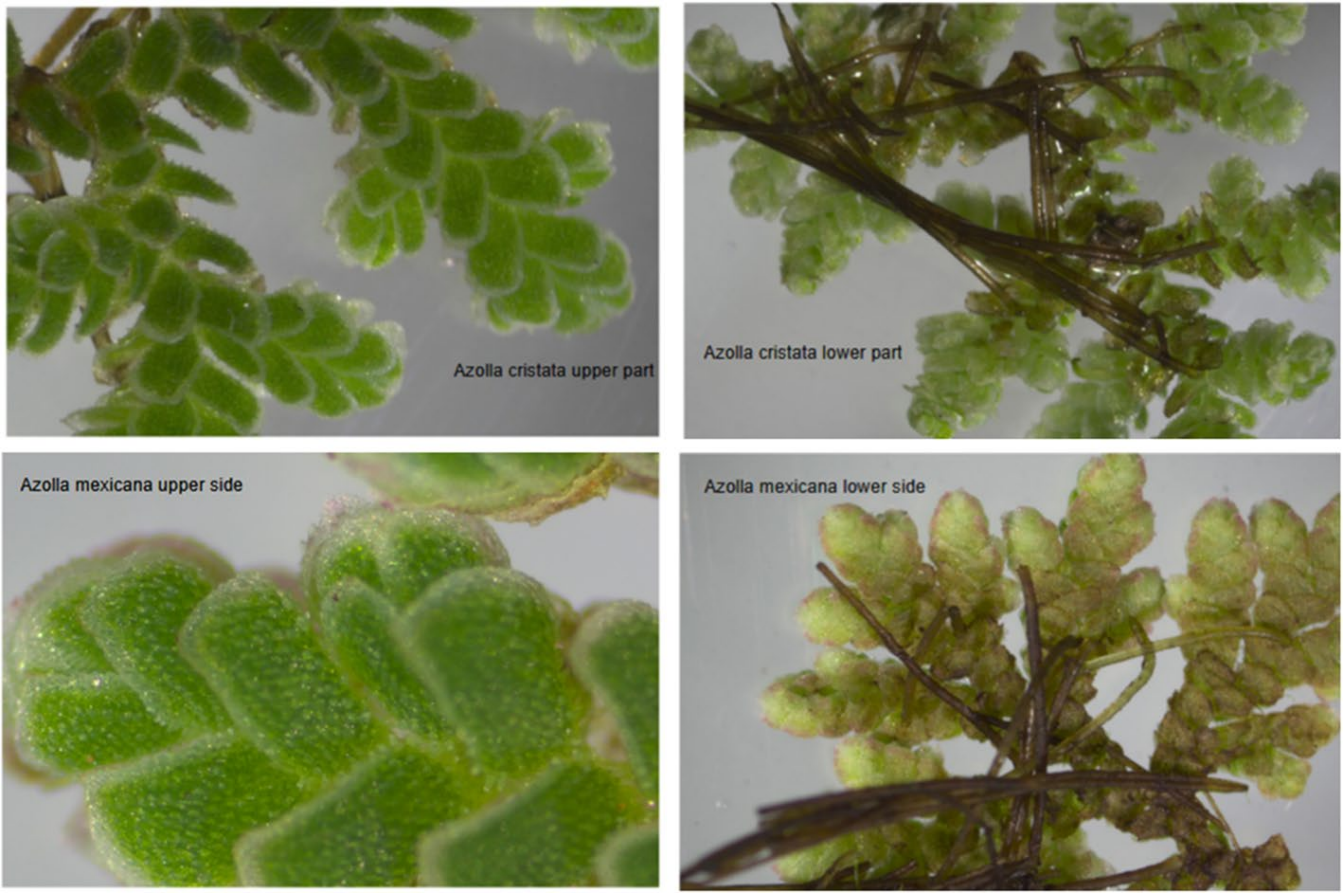
Morphological appearance of *Azolla cristata* and *Azolla mexicana*

*Azolla* samples collected and identified as different species, were visually examined under the dissecting microscope at X 60 magnification (Fig. 1). The major difference between *Azolla cristata* and the other *Azolla* species was that the former, exhibited a lime green appearance with colorless fronds while all the rest including *Azolla mexicana*, exhibited darker green leaves and a pinkish substance on the fronds (Fig. 1).

### Molecular characterization of *Azolla*

**Fig. 2:**
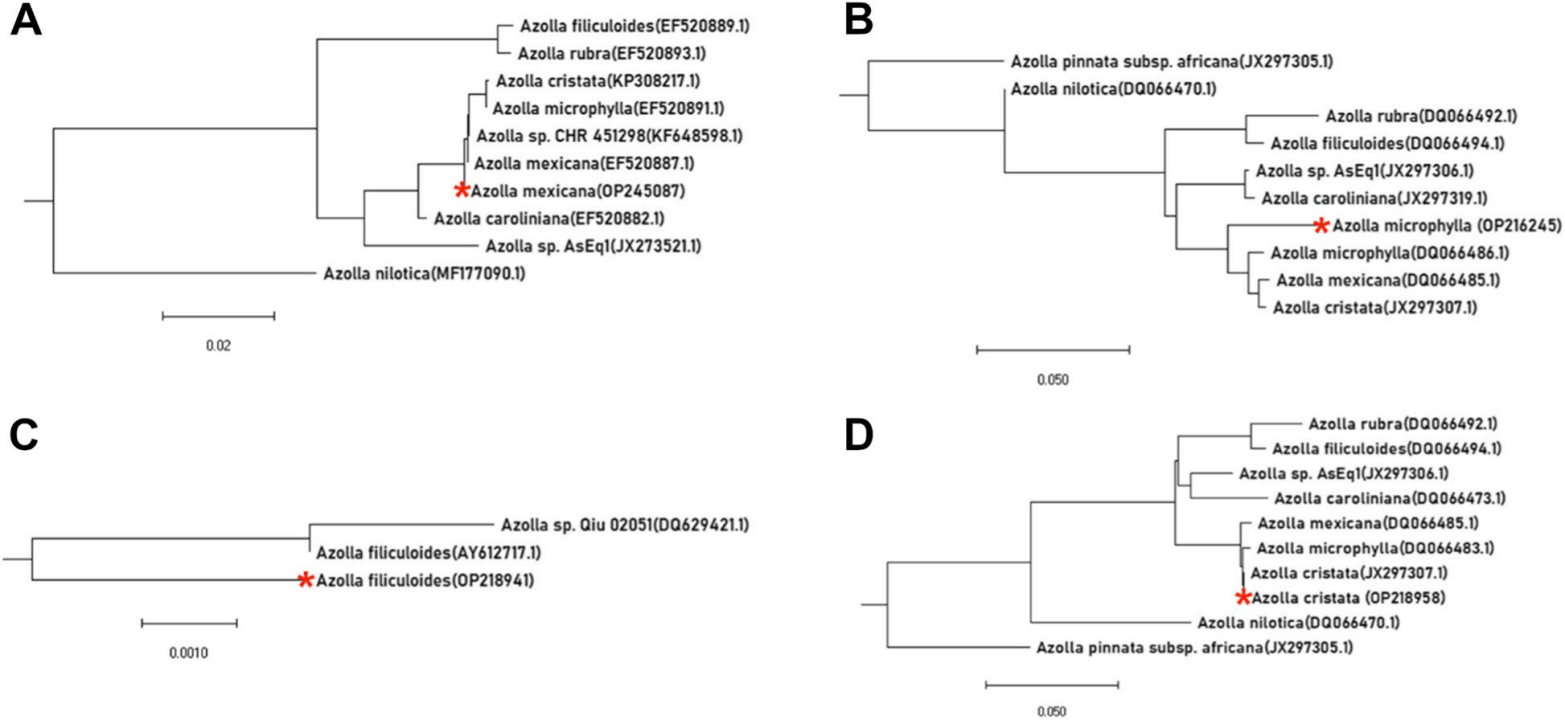
Phylogenetic trees showing the evolutionary relationships between the sample species under study and other database species

The target loci for molecular characterization of the *Azolla* species included; the ITS region, the trnL-trnF intergenic spacer and the region between the trnL gene and trnL-trnF intergenic spacer, utilizing different primers (Table 1). The PCR amplicons obtained were sequenced and the Sanger sequences for each species, were compared with the reference sequences in the NCBI database. Phylogenetic trees were generated following multiple sequence alignment with the reference database sequences (Fig. 2, − red star shows the *Azolla* species with the highest sequence identity to the sequences generated in this study).

**Table 1:** Primers for amplification of various loci from Azolla genomic DNA

Primer name	Primer sequence	Reference	Targeted locus	Sequence Length (bp)
ITS1 ITS4	5′ TCCGTAGGTGAACCTGCGG 3′ 5′ TCCTCCGCTTATTGATATGC 3′	[32]	Internal transcribed spacer 1, 5.8S ribosomal RNA, internal transcribed spacer 2	984
N-F N-R	5′ GGTTCAAGTCCCTCTATCCC 3′ 5′ ATTTGAACTGGTGACACGAG 3′	[26]	trnL – trnF intergenic spacer	409
SCAR-F SCAR-R	5′GACATATCCACCTATCGTCTCTGTC 3′ 5′AGACAACTTCGATAGTCACAGTTCC 3′	[1]	Internal transcribed spacer 1, 5.8S ribosomal RNA, internal transcribed spacer 2	842
18Sr-F 18Sr-R	5′ AGGGTTCGATTCCGGAGA 3′ 5′ CCTTCCGTCAATTCCTTTAAG 3‘	[6]	trnL gene and trnL-trnF intergenic spacer	1029

These generated sequences species were assigned new accession numbers after deposition in the NCBI Genbank repository as; OP245087, OP216245, OP218941 and OP218958 (Table 2). The new accessions showed 100, 93.36, 99.22 and 99.39% sequence identities to *Azolla mexicana* (Fig. 2A), *Azolla microphylla* (Fig. 2B), *Azolla filiculoides* (Fig. 2C) and *Azolla cristata* (Fig. 2D)*,* respectively.

**Table 2:** Distribution of *Azolla* species in the ten agro-ecological zones of Uganda

No.	Agro-ecological zone	*Azolla* species identified
1.	Kyoga plains	*A. cristata*
2.	Lake Victoria Crescent	*A. mexicana, A. microphylla, A. cristata*
3.	Para Savannah	*A. Mexicana*
4.	Western savannah grasslands	*A. microphylla, A. filiculoides, A. cristata*
5.	North Eastern drylands	None
6.	Highland Ranges	None
7.	North Eastern savannah grasslands	None
8.	North Western savannah grasslands	None
9.	Pastoral rangelands	None
10.	South Western Farmlands	None

### Ecological distribution

**Fig. 3:**
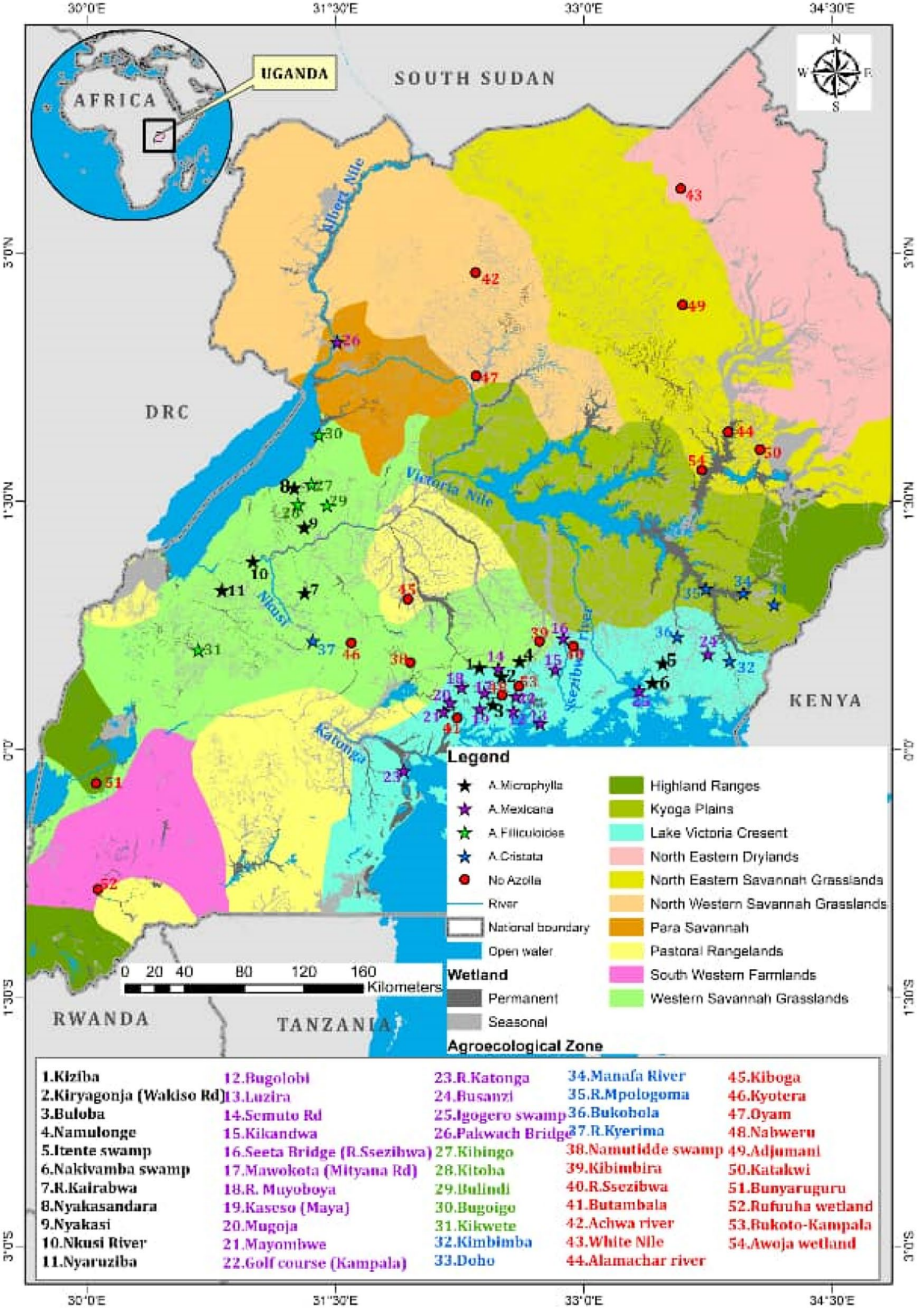
Distribution of Azolla species in the ten agro-ecological zones of Uganda

The map (Fig. 3) illustrates how *Azolla* was distributed within the ten agro-ecological zones of Uganda at the time of the survey and sample collection. From the map it is absolutely clear that *Azolla* species were distributed in wetlands of only four agro-ecological zones which were in close vicinity to large water masses (Fig. 3 and Table 3). Despite scanning all the ten agro-ecological zones, no traces of *Azolla* were identified in any of the remaining six zones.

**Table 3:** Voucher numbers of *Azolla* specimen samples deposited at the Makerere University Herbarium and accession numbers of *Azolla* sequences provided by the NCBI GenBank

Taxon	Herbarium Voucher ID	GenBank accession no.
*Azolla microphylla*	MUK:PMB:NLF:1	OP216245
*Azolla mexicana*	MUK:PMB:NLF:2	OP245087
*Azolla cristata*	MUK:PMB:NLF:3	OP218958
*Azolla filiculoides*	MUK:PMB:NLF:4	OP218941

### Environmental influences on the distribution of *Azolla* in Uganda

**Fig. 4:**
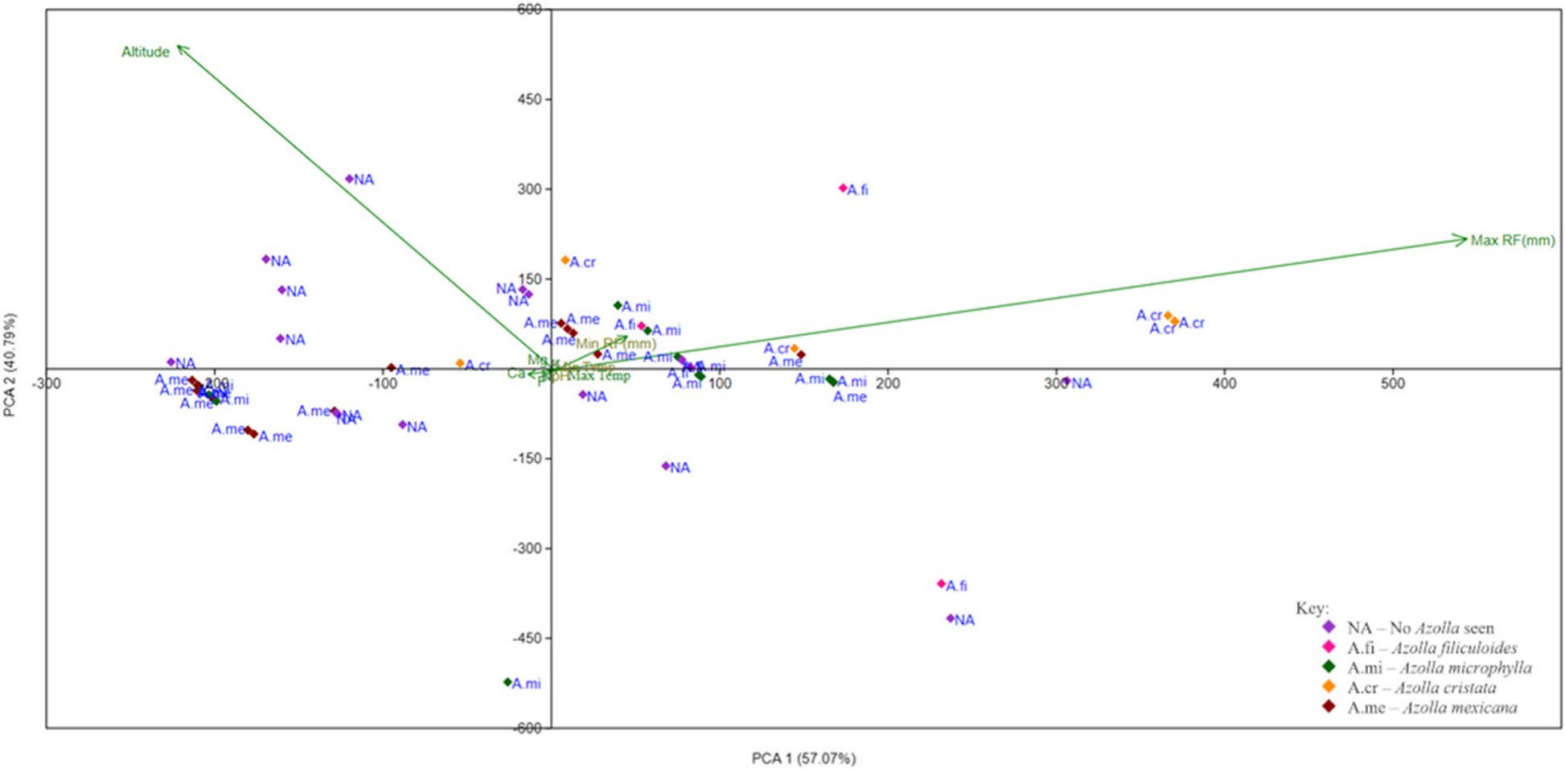
PCA biplot of Azolla species on the mineral nutrients, pH, altitude and climatic parameters

Data on pH and mineral nutrient concentrations (Ca, Mg, N, P and K) in water samples was analyzed together with altitude and annual average climatic data (rainfall and temperature), to determine their influence on the distribution of *Azolla* species in Uganda (Fig. 4).

The PCA biplot, demonstrated that only maximum rainfall and altitude with factor loadings of 0.921 and 0.922, respectively influenced the distribution of *Azolla*. All other factors exhibited no significant influence as supported by factor loadings of < 0.01. This signified the existence of other underlying factors that probably influenced this distribution.

## Discussion

### Molecular characterization of *Azolla*

The high percentage identity of the four sequences aligned with the reference sequences indicated a very close linkage to the database species. Based on these results, it was confirmed that the sample species were similar to the known database species. Thus, it was deduced that four species of *Azolla* namely; *A. mexicana, A. microphylla, A. filiculoides* and *A. cristata* existed in Uganda at the time of this study.

According to [30] two species namely; *Azolla pinnata* and *Azolla nilotica* were earlier identified from Lake Victoria regions and other parts of Uganda using morphological methods. According to [29], *Azolla pinnata* was identified on Nyamuriro wetland in Kigezi and Bulamogi in Kamuli district along Lake Kyoga near the floating papyrus vegetation. Other places included along Lake George in Western Uganda, Jinja -Tororo road, Mpigi swamp in Central region, and Bukoto county in Masaka district*. Azolla nilotica* was found in Queen Elizabeth National Park, Namanve swamp along R. Nile, Ssese islands and in West Nile region along R. Nile. Despite these past discoveries, this study did not identify any of the two species as discovered earlier. According to [10] morphological, physiological and reproductive structural identification of *Azolla* species is still difficult and unclear. Basing on this argument, and the method of identification employed at that time, this scenario creates suspicions of a probable misapplication of names. To further strengthen this argument, according to [12], there was a study conducted on a biological control experiment to eliminate *Azolla pinnata* subsp. Africana using *Stenopelmus rufinasus* (a water fern weevil). During this experiment, it was observed that *Azolla pinnata* was not the right host for *Stenopelmus rufinasus.* On conducting further analysis on *Azolla pinnata* to determine its true identity using molecular tools, it was proven that this species was instead *Azolla cristata* and not *Azolla pinnata*. This implied that the failure of that experiment was due to employment of a wrong biological control agent. More so, from this study, some of the agro-ecological zones where *Azolla cristata* was identified are synonymous with the regions where *Azolla pinnata* was earlier discovered. Further still, from the general appearance of *Azolla pinnata* as per the Makerere University Herbarium specimen archives, its fronds present a dark green appearance with a pinkish component (Fig. 1) most likely due to anthocyanin production. Since *Azolla mexicana, Azolla microphylla* and *Azolla filiculoides* all present a similar appearance, it could probably be assumed that *Azolla pinnata* identified earlier was actually one of the three above species. *Azolla cristata* instead, exhibits a lime green appearance with colorless fronds (Fig. 1). It is not clear whether *Azolla cristata* existed earlier since there are no records of its discovery as per the University Herbarium archives. Thus, it could be assumed as a new species that colonized these different regions. The new species could probably have been introduced indirectly by aquatic fowls and flowing water or even directly by man.

Despite having been documented as earlier discovered, *Azolla nilotica* was not identified anywhere as per this study. It is also unclear whether it was as well due to misapplication of a name or extinction the species. The specimens of *Azolla nilotica* as per the Herbarium archives exhibit a unique size of *Azolla* which was never observed in this study. Based on its unique features especially the size, there was no species among the four which appeared similar to it. This implies that it probably neither existed at the time of this study nor was it a misapplication of a name. Nonetheless, it is plausible that *Azolla nilotica* was out-competed by the onset of *Eichornia crassipes* (water hyacinth) on Lake Victoria. According to [15], the colonization of *Eichornia crassipes* pushed other macrophytes to the shores including *Azolla nilotica*, *pistia stratiotes*, *Vossia cuspidate* among others. Being smaller than other macrophytes, *Azolla nilotica* formed a substrate mass which later provided nutrients for growth of other competing macrophytes. Further still, the evolutionary fact of life steered by human pressures that created the unfavorable environment, coupled with climate change, [23] could also have contributed to the complete disappearance of *A. nilotica* from those regions.

### Ecological distribution of *Azolla*

From the PCA biplot (Fig. 4), only maximum rainfall and altitude showed a significant influence on the distribution of *Azolla*. The factor of maximum rainfall could be explained by the tendency of heavy rains to supply lots of water. With an overflow from large water masses into surrounding areas and swamps, a water-logged environment is created enabling *Azolla*’s growth and continuous distribution as water flows. An example is in the Para Savannah agro-ecological zone along Albert Nile, where *A. mexicana* was found stuck in the nearby vegetation. This implies that it was probably being carried along with the flowing water but remained stuck and was later identified there. Further still, distribution could also be by aquatic fowls and other organisms [31] as they move from place to place carrying spores alongside. These spores can be deposited anywhere and if conditions turn out favorable, they germinate forming mature plants.

A high altitude negatively influenced the distribution of *Azolla* according to the PCA results. This could be attributed to the reduced water levels, a factor that does not support growth of *Azolla*. Further still, at high altitude, there is an increase in exposure to environmental stresses that affect species distribution. Among these are the reduced levels of micronutrients arising from low mineralization thereby, lowering the pH [8].

Topography was another factor that probably contributed to the absence of *Azolla*, in the Highland ranges and the South Western farmlands of Uganda. *Azolla* requires conditions of no or very slow-moving water to thrive. However, in these two agro-ecological zones, the nature of the land is mountainous thus the water flow coming down from the hills is always very fast [28]. This condition hinders the growth and survival *Azolla*.

The North Eastern drylands, North Western Savannah grasslands, North Eastern Savannah grasslands and the Pastoral rangelands mostly possess seasonal wetlands with animal grazing as the main activity [28]. Most of the swamps that were scanned in these regions were contaminated with unclean water from disruption by drinking animals coupled with waste dumping. The prolonged disturbance causing consistent turbulence of water hinders *Azolla*’s growth and survival [23]. This probably explains its absence in those regions.

Population pressure was another observed factor that affected *Azolla*’s growth, survival and distribution. Uganda is a country with several wetlands. However, owing to population growth coupled with the scarcity of land, wetlands which constitute the major habitat for *Azolla* have been severely encroached upon. The egocentric character of man coupled with ignorance about the benefits of *Azolla* account for the massive clearance of its habitats. This is conducted in favor of several activities including agriculture, bricklaying, construction of settlements, fishing and animal grazing [28]. Despite all trials for its elimination, *Azolla* is still able to regenerate when favorable conditions are availed due to its invasive nature thus enabling its persistence in the environment.

The mineral nutrients analyzed were expected to have a significant influence on the distribution of *Azolla*, since they are among the major macronutrients required for growth of plants. However, PCA results showed no significant influence, an issue that could probably be attributed to the constantly fluctuating mineral nutrient concentrations in the wild environment.

From the map (Fig. 3) *Azolla* was identified in four out of the ten agro-ecological zones of Uganda. Despite this, there is no clear evidence that *Azolla* exists only in these areas. *Azolla* is known to grow and thrive under specific conditions including temperature between 18 and 25 °C, a shady environment with low light intensity, pH 4.5 – 7.5 and humidity of 70-75% [23]. When conditions become extremely unfavorable it may die and its spores regenerate later when appropriate conditions return. This explains why in some areas where it was collected, its color was turning from green to reddish brown. This change is due to production of more anthocyanins and less chlorophyll following environmental stresses like elevated temperatures [9, 34]. However, when appropriate conditions resume, more chlorophyll is produced with less anthocyanins stimulating the regeneration of the green color.

## Conclusion

This study identified four different species of *Azolla* namely; *Azolla Mexicana, Azolla microphylla*, *Azolla filiculoides,* and *Azolla cristata*. These species were majorly identified in four agro-ecological zones including Lake Victoria crescent, Kyoga plains, Para Savannah and Western Savannah grasslands which are in close vicinity to large water masses. The only two species which had been discovered previously were not identified in this study. This implied that it was either a misapplication of a name, replacement by new species or probably out competition by other macrophytes, as for the sake of *Azolla nilotica*. Maximum rainfall significantly influenced the growth of *Azolla* unlike a high altitude. The major observed factors that affected the growth and distribution of *Azolla* were anthropogenic factors which enhance turbulence and favor ecosystem disturbance. The other factor was topography characterized by hilly and mountainous areas that cannot favor smooth and slow-moving water required for the survival of *Azolla*.

### Recommendation

There is a need to develop standard methods of preserving *Azolla* for future use, research and reference considering the rate of wetland clearance in Uganda. Further still, investigations on the two unidentified species during the study; i.e. *A. pinnata* and *A. nilotica* need to be made to confirm whether it was a taxonomic name misapplication or actual disappearance of species resulting from several causes. More studies on the ecological distribution of the different species of *Azolla* in Uganda should be conducted, to determine the factors influencing their existence in a location over a reasonable period of time.

## Materials and methods

### Scope of the study

Uganda is a landlocked country lying within the equatorial region with a latitude of 1.3733° N, and a longitude of 32.2903° E. It occupies 241,550.7 Km^2^ of land, with open water and swamps constituting 41,743.2 Km^2^ [14]. Most of these water bodies are supplied by rainfall. Some rivers drain into wetlands while other wetlands arise from underground water trickling out of aquifers.

### Study area

Agro-ecological zones characterized by wetlands with slow moving waters and drained with waters from different major rivers in Uganda were selected for the study to obtain a representative sample for the entire country. Both permanent and seasonal wetlands were considered as part of the sampling zones. These agro-ecological zones included; Highland ranges, Kyoga plains, Lake Victoria Crescent, North Eastern drylands, North Eastern Savannah grasslands, North Western Savannah grasslands, Para Savannah, Pastoral ranges, South Western farmlands, and Western Savannah grasslands [16]. The study was registered and approved by the National Council of Science and Technology with an approval number of UNCST/RCI/94811239. Permission to carry out a country wide survey in search of *Azolla* was also provided.

### Sampling procedure

During sampling, wetlands within the same hydrological region drained by the same river were assumed to be harboring the same species of *Azolla* and were thus partially scanned. Because *Azolla* is an aquatic plant, purposive sampling was done mainly from wetlands of different agro-ecological zones to obtain representative samples for a particular area. Majority of the sites where *Azolla* was collected were characterized by water-logged clay soils with papyrus as the dominant vegetation forming a swampy environment. Other places included ponds that had been abandoned after bricklaying, while others were plantations in wetlands as well as some shore zones of weedy channels that allow limited water flow. *Azolla* was collected during the rainy season in the months of November 2019, January, March, August, and November 2020. The sites where *Azolla* was collected ranged between 680 and 1308 m above sea level although there were still other sites within the same altitude where *Azolla* was not found.

*Azolla* specimens were initially identified by a senior taxonomist Mr. Rwabulindooli from the Makerere University herbarium. After confirming that the material sampled was *Azolla*, massive sample collection commenced. The collected *Azolla* samples were packed in well labelled 50 ml falcon tubes and transferred into a cold box maintained at 4 °C. These were then transported to the Molecular diagnostic laboratory in the Department of Plant Sciences, Microbiology, and Biotechnology at Makerere University. From the same spot where *Azolla* was collected, a corresponding water sample was collected into a well labelled water sample bottle, transferred into a cold box to the Geochemistry laboratory at Makerere University for mineral nutrient analysis.

### Morphological characterization of the *Azolla* samples

*Azolla mexicana* having had a similar appearance to *Azolla microphylla* and *Azolla filiculoides* was selected for morphological characterization in comparison with *Azolla cristata.* A section of these was mounted on to a slide of a dissecting microscope connected to the computer and the image viewed under X60 magnification.

### Chemical analysis of the water samples

Water was analyzed for the presence of dissolved calcium, phosphorus, nitrogen, potassium, and magnesium using the Atomic Absorption Spectrophotometer (AAS) [17].

### DNA extraction

The samples of *Azolla* stored at 4 °C were retrieved after 1 day. About 200 mg of *Azolla* tissue was first cleaned with distilled water and ground into a fine powder using a sterile mortar and pestle under liquid nitrogen. DNA extraction was carried out using the Bioron Plant DNA Mini Kit according to Manufacturer’s instructions (BIORON Diagnostics GmbH, Römerberg, Germany). The concentration and quality of the extracted DNA were determined using 1% gel electrophoresis and a NanoDrop One C spectrophotometer (Thermo Fisher Scientific, Madison, USA). The isolated genomic DNA was then stored at − 20 °C until use.

### DNA amplification and sequencing

In this study, DNA amplification was successfully conducted using four different primer sets (Table 1). The PCR product was prepared in a total volume of 25 μl containing One Taq Quick-load 2X Master Mix (New England BioLabs Inc., Cat.No.: MO486S), 0.2 μM of each primer and 250 ng/μl of DNA template or same concentrations of Q5 Hot start High Fidelity Master Mix from (New England Biolabs, USA) for difficult amplifications. The mixture was topped up to 25 μl with nuclease-free water. Amplification was carried out in an XP Thermal cycler (Hangzhou Bioer Technology Co, Ltd., Hangzhou, China). All reactions were performed for 35 cycles with the following conditions; Initial denaturation at 94 °C for 3 minutes and 20 seconds, denaturation at 94 °C for 25 seconds, annealing at 53 °C for 30 seconds, extension at 72 °C for 50 seconds and the final extension at 72 °C for 5 minutes.

### Gel electrophoresis

The amplification success was checked by 1.5% agarose gel stained with ethidium bromide. Electrophoresis was carried out for 1[1]/_2_ hours at 100 V with a current of 300 A. Gel images were then viewed under a UV trans illuminator (Model: PS300TP, Taiwan).

### Sanger sequencing

PCR products were purified and sequenced by ‘Intergen Genetics and Rare Diseases Diagnosis and Research Centre’ in Turkey using standard operating procedures.

### Phylogenetic analysis

Sequences were edited using the BioEdit biological sequence alignment editor version 7.2 [Hall, 1999]. Basic Local Alignment Search Tool (BLAST) searches were applied to all produced sequences using National Centre for Biotechnology Information (NCBI/GenBank) databases. A megablast was conducted (Nucleotide BLAST) with highly similar sequences considered [2]. At genus level, identification was considered as successful when all hits with maximal percentage identity scores > 98% involved a single genus. Species identification was considered successful only when the highest maximal percentage identity included a single species and scored > 90% [33]. All sequences were matched with the query sequences and available sequences of *Azolla* in order to determine the best matching species. The phylogenetic trees were generated using blast pairwise alignment and the sample sequences were aligned with the database sequences. The final images were downloaded into a Newick file for editing into final scaled phylogenetic trees using Molecular Evolutionary Genetics Analysis version 11 (MEGA 11) software [27].

### Specimen deposition in the university herbarium and NCBI GenBank

After confirmation of species with molecular tools, four specimens corresponding to the four different species were deposited in the Makerere University Herbarium where they were assigned voucher numbers by Mr. Rwabulondooli Protase, a senior taxonomist in the University. The four species’ sequence data was also submitted to NCBI GenBank repository and accession numbers were assigned.

### Environmental conditions for the sample collection sites

Data on pH, altitude and average annual climate (temperature and rainfall) was collected between 2011 and 2021 and analyzed.

### Data analysis

To determine the influence of the parameters under study (mineral nutrients, pH, altitude and climatic data) on the distribution of *Azolla* in different agro-ecological zones, the principal component analysis (PCA) was computed using PAST [7].

## Data Availability

Sequence datasets generated and analyzed during the current study were deposited in the NCBI GenBank repository and assigned accession numbers as; OP245087, OP216245, OP218941, and OP218958. These are currently publicly available when searched using the following links. https://www.ncbi.nlm.nih.gov/nuccore/OP216245.1https://www.ncbi.nlm.nih.gov/nuccore/OP218941.1https://www.ncbi.nlm.nih.gov/nuccore/OP218958.1https://www.ncbi.nlm.nih.gov/nuccore/OP245087.1 The other datasets used during the current study are available from the corresponding author on reasonable request.
